# Updated Recommendation for Universal Hepatitis B Vaccination in Adults Aged 19–59 Years — United States, 2024

**DOI:** 10.15585/mmwr.mm7348a3

**Published:** 2024-12-05

**Authors:** Amy L. Sandul, Karina Rapposelli, Melissa Nyendak, Min Kim

**Affiliations:** 1 Division of Viral Hepatitis, National Center for HIV, Viral Hepatitis, STD, and TB Prevention, CDC.

**Summary**

**What is already known about this topic?**

Vaccination is the principal means for preventing hepatitis B virus infection. Hepatitis B (HepB) vaccines are safe and effective. Advisory Committee on Immunization Practices recommendations include HepB vaccination of all adults aged 19–59 years, including pregnant persons. Pregnant persons may receive any HepB vaccine licensed for adults for which data are sufficient to evaluate vaccine-associated risks in pregnancy.

**What is added by this report?**

On September 11, 2024, the Food and Drug Administration approved updates to the package insert for Heplisav-B [HepB vaccine (recombinant), adjuvanted], Section 8.1 (Pregnancy) to include human data that do not suggest an increased risk for both major birth defects and miscarriage.

**What are the implications for public health practice?**

Providers can now administer Engerix-B, Heplisav-B, Recombivax HB, or Twinrix to pregnant persons needing HepB vaccination.

Hepatitis B (HepB) vaccines have demonstrated safety, immunogenicity, and efficacy during the past 4 decades (*1*,*2*). The Advisory Committee on Immunization Practices recommends universal HepB vaccination for adults aged 19–59 years, including pregnant persons, and adults aged ≥60 years with risk factors for hepatitis B. Adults aged ≥60 years without known risk factors for hepatitis B may also receive HepB vaccines (*2*).

On September 11, 2024, the Food and Drug Administration approved a request to update the labeling for Heplisav-B vaccine with new indications for use among pregnant persons (*3*,*4*). A postlicensure observational retrospective cohort study (DV2-HBV-28)* included 75 pregnancies with known outcomes, including 10 among persons who received Heplisav-B twice during the period from 28 days before conception through the end of pregnancy. Among 75 pregnant persons with exposure to Heplisav-B before or during pregnancy, 44 received Heplisav-B during the 28 days before conception, 24 during the first trimester, six during the second trimester, and one during the third trimester. No major birth defects were identified, and the risk for miscarriage was below the estimated background risk. These available data, primarily for persons who received 1 dose of Heplisav-B during the 28 days before conception, or during pregnancy, do not suggest an increased risk for both major birth defects and miscarriage. Approval by the Food and Drug Administration under section 351(a) of the Public Health Service Act^†^ for Hepatitis B Vaccine (Recombinant), Adjuvanted (HEPLISAV-B), to update the package insert to include data from study DV2-HBV-28, allows for use of Heplisav-B to vaccinate pregnant persons needing HepB vaccination (*3*,*4*).

## Recommendation

Providers should vaccinate pregnant persons needing HepB vaccination with Engerix-B,^§^ Heplisav-B,^¶^ Recombivax HB,** or Twinrix.^††^

## Footnotes

*
https://catalogues.ema.europa.eu/node/4205/methodological-aspects

†
https://uscode.house.gov/view.xhtml?req=(title:42%20section:262%20edition:prelim)

§
https://www.fda.gov/media/119403/download

¶
https://www.fda.gov/media/108745/download

**
https://www.fda.gov/files/vaccines%2C%20blood%20%26%20biologics/published/package-insert-recombivax-hb.pdf

††
https://gskpro.com/content/dam/global/hcpportal/en_US/Prescribing_Information/Twinrix/pdf/TWINRIX.PDF

## References

[1] Schillie S, Vellozzi C, Reingold A, Prevention of hepatitis B virus infection in the United States: recommendations of the Advisory Committee on Immunization Practices. MMWR Recomm Rep 2018;67:1–31 10.15585/mmwr.rr6701a1 29939980 PMC5837403

[2] Weng MK, Doshani M, Khan MA, ; CDC. Universal hepatitis B vaccination in adults aged 19–59 years: updated recommendations of the Advisory Committee on Immunization Practices—United States, 2022. MMWR Morb Mortal Wkly Rep 2022;71:477–83. 10.15585/mmwr.mm7113a1 35358162 PMC8979596

[3] Food and Drug Administration. Supplement approval [Letter]. Silver Spring, MD: US Department of Health and Human Services, Food and Drug Administration; 2024. https://www.fda.gov/media/181781/download

[4] Food and Drug Administration. HEPLISAV-B [package insert]. Silver Spring, MD: US Department of Health and Human Services, Food and Drug Administration; 2024. https://www.fda.gov/vaccines-blood-biologics/vaccines/heplisav-b

